# Supplementary material for: Content-rich biological network constructed by mining PubMed abstracts
Source: BMC Bioinformatics. 2004 Oct 8;5:147. doi: 10.1186/1471-2105-5-147 (PMC528731; doi:10.1186/1471-2105-5-147)
Supplement: Additional File 2 — The original results of the above study (non-essential files are deleted to keep the file size under the limit set by BMC bioinformatics). [file 1471-2105-5-147-S2.bz2 › chilibotAdditionalFile2/dip30/427ID11470772E10357/html/CCL2_CCR2.html]

 


 **CCL2** and **CCR2** 
  
Found 2851 abstracts in PubMed, retrieved 30.  
 

 What does Google say? 
 PDF only 
| .edu only 

---

**Interactive relationship** (e.g. stimulation, inhibition, etc)

**Non-interactive relationship** (e.g. studied together, co-existance, homology, etc.)

- Increased concentrations of cytokines, IL 6, IL 8, and MCP 1  [ **CCR2** ]   [ **CCL2** ] , were detected in the supernatant of cocultures of HUVEC with biomaterial treated whole blood or mononuclear cells but not neutrophils or platelets, compared with the media control.  Ref: 12870471 J Biomed Mater Res, 2003
- Concerning the other cytokines and chemokines studied MCP 1  [ **CCR2** ]   [ **CCL2** ] , IL 8 and IL 12, no significant differences were found to be associated with the radiological profiles.  Ref: 12872499 G Ital Med Lav ErgonG Ital Med Lav Ergon, 2003
- Indeed, we find that RP3 alone causes increases in nuclear NF kappaB activity and secretion of MCP 1  [ **CCR2** ]   [ **CCL2** ]  and GM CSF.  Ref: 12881713 Oncogene, 2003
- increased CD40 ligand induced MCP 1  [ **CCR2** ]   [ **CCL2** ]  production.  Ref: 12874180 CirculationCirculation, 2003
- We detected an upregulation or de novo synthesis of interleukin 1alpha IL 1alpha, IL 1beta, IL 8, macrophage inflammatory protein 1alpha MIP 1alpha, MIP 1beta, GRO alpha, transforming growth factor beta, MCP 1  [ **CCR2** ]   [ **CCL2** ] , IL 2 receptor gamma IL 2Rgamma, IL 10Ralpha, and IL 6R.  Ref: 12874345 Infect Immun, 2003
- IL 6, MIP 2 and MCP 1  [ **CCR2** ]   [ **CCL2** ]  appear to follow the toxicity.  Ref: 12883092 Toxicol SciToxicol Sci, 2003
- In addition, MCP 1  [ **CCR2** ]   [ **CCL2** ]  production of PPD or 30 kDa antigen stimulated monocytes was significantly elevated in CR TB patients than that from E TB.  Ref: 12861395 Inflamm Res, 2003
- DESIGN AND PATIENTS Immunocytochemistry and reverse transcriptase polymerase chain reaction RT PCR amplification were used to analyse the expression of monocyte chemoattractant protein MCP 1  [ **CCR2** ]   [ **CCL2** ] , RANTES regulated upon activation, normal T cell expressed and secreted, macrophage inflammatory protein MIP 1alpha, MIP 1beta, interferon IFN gamma inducible protein IP 10 and monokine induced by IFN gamma Mig in thyroid tissue from patients with Hashimoto s thyroiditis n = 4, Graves disease n = 6 and nonautoimmune multinodular goitre n = 4.  Ref: 12864798 Clin Endocrinol (Oxf), 2003
- CONCLUSIONS The data suggest that MCP 1  [ **CCR2** ]   [ **CCL2** ]  production is not uniquely elevated systemically in TB patients, although MCP 1  [ **CCR2** ]   [ **CCL2** ]  production might be elevated by monocytes in the chronic phase of TB or with a local pleural infection.  Ref: 12861395 Inflamm Res, 2003
- After 24 h, cytokine release was significantly increased for both IL 8 and MCP 1  [ **CCR2** ]   [ **CCL2** ]  but not IL 6 above concentrations after 4 h of coculture.  Ref: 12870471 J Biomed Mater Res, 2003
- Monocyte chemoattractant protein 1  [ **CCL2** ]  MCP 1  [ **CCR2** ]   [ **CCL2** ]  and interleukin 8 IL 8 are major chemokines for leukocyte trafficking and have been identified in atheromatous plaques.  Ref: 12881478 Circ ResCirc Res, 2003
- Monocyte chemoattractant protein MCP 1  [ **CCR2** ]   [ **CCL2** ]  and macrophage colony stimulating factor M CSF are two of the associated chemokines, but their role in coronary artery disease has not been sufficiently clarified.  Ref: 12892039 J Cardiol, 2003
- We found that clinically relevant levels of Hcy 10 to 1000 micro mol L increased the protein secretion and mRNA expression as well as activity of MCP 1  [ **CCR2** ]   [ **CCL2** ]  and IL 8 in cultured primary human monocytes.  Ref: 12881478 Circ ResCirc Res, 2003
- Five genes were expressed at lower levels in unstable angina samples anticoagulant protein S, cyclooxygenase COX 1, interleukin IL 7 and chemokines monocyte chemotactic protein MCP 1  [ **CCR2** ]   [ **CCL2** ]  and 2.  Ref: 12871422 J Thromb Haemost, 2003
- Although no associations were found in alleles or genotypes, MCP 1  [ **CCR2** ]   [ **CCL2** ]  2518A G genotype affected the age at onset of PD patients.  Ref: 12889089 Mov Disord, 2003
- For monocytes, CR TB patients spontaneously expressed more MCP 1  [ **CCR2** ]   [ **CCL2** ] , compared with HTRs and E TB patients.  Ref: 12861395 Inflamm Res, 2003
- The MCP 1  [ **CCR2** ]   [ **CCL2** ]  protein level in the cortical ischemic cores of the normothermic subgroups began to increase since the 6th hour afer reperfusion 22.5 8.7 ng.g tissue 7 times that in the sham operation samples, P <.  Ref: 12887739 Zhonghua Yi Xue Za Zhi, 2003
- Groups of rats were exposed to hypoxic and hyperoxic environmental conditions for 60 h and MCP 1  [ **CCR2** ]   [ **CCL2** ]  was determined in their peripheral blood mononuclear cells by Elisa and Reverse Transcriptase Polymerase Chain Reaction RT PCR.  Ref: 12870648 Mol Cell Biochem, 2003
- Sustained shear stress downregulates atherogenic genes e.g., MCP 1  [ **CCR2** ]   [ **CCL2** ]  and the genes that facilitate lipid accumulation and upregulates growth arrest genes.  Ref: 12865076 Prog Biophys Mol Biol, 2003
- Whereas treatment with infectious virus induced a rapid down regulation of the  **CCL2**  monocyte chemoattractant protein 1  [ **CCL2** ]  MCP 1  [ **CCR2** ]   [ **CCL2** ]  specific receptor  **CCR2** , inactivated virus did not significantly alter  **CCR2**  surface expression.  Ref: 12885942 J Leukoc Biol, 2003
- Because MCP 1  [ **CCR2** ]   [ **CCL2** ]  induces monocyte immigration and differentiation to macrophages, which augment extracellular matrix production and tubulointerstitial fibrosis, pharmacological reduction of angiotensin II may also exert its beneficial effects in diabetic nephropathy by downregulation of renal MCP 1  [ **CCR2** ]   [ **CCL2** ] .  Ref: 12882873 Diabetes Care, 2003
- ... TNF alpha induced a significant upregulation in ICAM 1 expression and in MCP 1  [ **CCR2** ]   [ **CCL2** ] , eotaxin and IL 6 release p< ; 0.05, each comparison.  Ref: 12877820 Pulm Pharmacol Ther, 2003
- CONCLUSION Mild hypothermia reduces the level of MCP 1  [ **CCR2** ]   [ **CCL2** ]  in the cortex after cerebral ischemia reperfusion which may be one of the important mechanisms of the neuroprotective effects of mild hypothermia.  Ref: 12887739 Zhonghua Yi Xue Za Zhi, 2003
- The MCP 1  [ **CCR2** ]   [ **CCL2** ]  levels were measured by enzyme linked immunosorbent assay ELISA.  Ref: 12861395 Inflamm Res, 2003
- NAC 10 mM decreased the expression of eotaxin and MCP 1  [ **CCR2** ]   [ **CCL2** ] , by 46 11% n=7 and 87 4% n=6, respectively.  Ref: 12882449 Eur Respir J, 2003
- and weak for IL 1beta and monocyte chemotactic peptide 1 MCP 1  [ **CCR2** ]   [ **CCL2** ] .  Ref: 12874304 Infect Immun, 2003
- ... IL 6, MIP 2 and MCP 1  [ **CCR2** ]   [ **CCL2** ]  were increased by 4 8 hours.  Ref: 12883092 Toxicol SciToxicol Sci, 2003
- This study investigated the expression of MCP 1  [ **CCR2** ]   [ **CCL2** ]  and c fms M CSF receptor in human coronary tissues.  Ref: 12892039 J Cardiol, 2003
- However, the M CSF c fms system, rather than MCP 1  [ **CCR2** ]   [ **CCL2** ] , is more important in the late stage of restenosis.  Ref: 12892039 J Cardiol, 2003
- In a mouse macrophage like cell line, the same beta isoform activation increases the release of the MCP 1  [ **CCR2** ]   [ **CCL2** ]  chemokine.  Ref: 12892998 Mol Aspects MedMol Aspects Med, 2003
- CONCLUSION On the basis of previous studies and the present case, the high production of IL 8 and MCP 1  [ **CCR2** ]   [ **CCL2** ]  by monocytes in LPS stimulated WBCC from periodontitis patients is most likely acquired, as their levels decrease over time when the periodontal infection is controlled.  Ref: 12887346 J Clin PeriodontolJ Clin Periodontol, 2003
- Monocyte chemotactic protein 1  [ **CCL2** ]  MCP 1  [ **CCR2** ]   [ **CCL2** ] , an important chemotactic cytokine can be activated by active oxygen species.  Ref: 12870648 Mol Cell Biochem, 2003
- Homocysteine stimulated MCP 1  [ **CCR2** ]   [ **CCL2** ]  mRNA expression and protein production in a time dependent and dose dependent manner in endothelial cells, decreased intracellular glutathione GSH and protein thiol levels, as well as G6PDH activity and NADPH levels.  Ref: 12883331 J Cardiovasc Pharmacol, 2003
- Twenty five subjects 24 males and 1 female, mean age 57.4 years who have been exposed to asbestos underwent chest radiography, high resolution computed tomography HRCT of the chest, lung function tests and bronchoalveolar lavage BAL for evaluation of cell components total cell count, percentages of macrophages, lymphocytes, neutrophil and eosinophil granulocytes and the lymphocyte subpopulations CD3, CD4, CD8, CD19 and HLADR, soluble factors IL 8, IL 10, IL 12 and MCP 1  [ **CCR2** ]   [ **CCL2** ]  in the supernatant and concentration of asbestos fibre.  Ref: 12872499 G Ital Med Lav ErgonG Ital Med Lav Ergon, 2003
- We examined if blockade of the renin angiotensin system lowered urinary levels of the chemokine MCP 1  [ **CCR2** ]   [ **CCL2** ]  and correlated urinary MCP 1  [ **CCR2** ]   [ **CCL2** ]  uMCP 1 with parameters of renal function and glucose and lipid metabolism before and after 1 year of treatment with an ACE inhibitor.  Ref: 12882873 Diabetes Care, 2003
- MCP 1  [ **CCR2** ]   [ **CCL2** ]  expression and production in monocyte macrophages from rats at normoxic conditions was reduced in aged subjects.  Ref: 12870648 Mol Cell Biochem, 2003
- The possible connection between periodontitis and atherosclerosis through IL 8 and MCP 1  [ **CCR2** ]   [ **CCL2** ]  is discussed.  Ref: 12887346 J Clin PeriodontolJ Clin Periodontol, 2003
- OBJECTIVE To investigate the effect of intraischemic mild hypothermia on the protein levels of interleukin IL 1beta and monocyte chemoattractant protein MCP 1  [ **CCR2** ]   [ **CCL2** ]  in the ischemic core of rat cortex after transient focal cerebralischemia.  Ref: 12887739 Zhonghua Yi Xue Za Zhi, 2003
- Additionally, resistin treated cells showed increased expression of VCAM 1 and MCP 1  [ **CCR2** ]   [ **CCL2** ] , with concomitant reductions in TRAF 3 expression.  Ref: 12874180 CirculationCirculation, 2003
- In pleural effusions, MCP 1  [ **CCR2** ]   [ **CCL2** ]  levels were significantly higher in patients with TBP than in patients with CHF, but lower than in malignant effusions.  Ref: 12861395 Inflamm Res, 2003
- the expression of CD11b of leukocytes was significantly higher than that of the control groups P< ; 0.01 and P< ; 0.05 the expression of MCP 1  [ **CCR2** ]   [ **CCL2** ]  mRNA of myocardial tissue began to rise at R60, reaching the highest at R360 P< ; 0.01 the concentration of Ca 2 of the serum was significantly higher than that of the control groups P< ; 0.01 and P< ; 0.05.  Ref: 12881868 Zhejiang Da Xue Xue Bao Yi Xue Ban, 2003
- The release of the chemokines interleukin IL 8 and macrophage chemoattractant protein MCP 1  [ **CCR2** ]   [ **CCL2** ]  in the cultures decreased twofold over time.  Ref: 12887346 J Clin PeriodontolJ Clin Periodontol, 2003
- ... the MCP 1  [ **CCR2** ]   [ **CCL2** ]  release was decreased by 69 41% n=10.  Ref: 12882449 Eur Respir J, 2003
- The following primary antibodies were used anti MCP 1  [ **CCR2** ]   [ **CCL2** ] , anti c fms, anti macrophages and anti alpha smooth muscle actin.  Ref: 12892039 J Cardiol, 2003
- Expression patterns of MCP 1  [ **CCR2** ]   [ **CCL2** ]  and c fms exhibited no difference between the two lesion types.  Ref: 12892039 J Cardiol, 2003
- Therefore the current study investigated whether N acetylcysteine NAC, an antioxidative agent, inhibits the interleukin IL 1beta induced expression and production of eotaxin and monocyte chemotactic protein MCP 1  [ **CCR2** ]   [ **CCL2** ]  in human airway smooth muscle cells HASMC.  Ref: 12882449 Eur Respir J, 2003
- MgSO 4 may protect myocardial tissue from ischemic reperfusion injury by inhibiting the expression of Ps of platelets and MCP 1  [ **CCR2** ]   [ **CCL2** ]  mRNA of myocardial tissue and decreasing the concentration of Ca 2 of the serum.  Ref: 12881868 Zhejiang Da Xue Xue Bao Yi Xue Ban, 2003
- RESULTS In sera, the MCP 1  [ **CCR2** ]   [ **CCL2** ]  levels of TB patients were similar to those of HTRs.  Ref: 12861395 Inflamm Res, 2003
- Furthermore, the inhibitors of mitogen activated protein kinase p38 and extracellular signal regulated kinase 1 2 and nuclear factor kappaB or the activator of peroxisome proliferator activated receptor gamma PPARgamma significantly decreased Hcy induced MCP 1  [ **CCR2** ]   [ **CCL2** ]  and IL 8 secretion in these cells.  Ref: 12881478 Circ ResCirc Res, 2003
- MCP 1  [ **CCR2** ]   [ **CCL2** ]  and IL 8 have been found to express mainly by macrophages in human lesion.  Ref: 12881478 Circ ResCirc Res, 2003
- These results demonstrate that homocysteine can trigger overexpression of the MCP 1  [ **CCR2** ]   [ **CCL2** ]  gene by altering the intracellular redox status, suggesting that the homocysteine induced changes in the intracellular redox status play an important role in modulating the expression of MCP  [ **CCL2** ]  l in endothelial cells.  Ref: 12883331 J Cardiovasc Pharmacol, 2003
- Interestingly, the E TB patients had significantly depressed MCP 1  [ **CCR2** ]   [ **CCL2** ]  production by PBMCs in response to PPD or 30 kDa, compared with HTRs and CR TB patients.  Ref: 12861395 Inflamm Res, 2003
- CONCLUSION We postulate that TFC may play a role in the pathogenesis of autoimmune thyroid disease as they are able to express the chemokines MIP 1alpha, MIP 1beta, MCP 1  [ **CCR2** ]   [ **CCL2** ] , RANTES, IP 10 and Mig that would promote the infiltration of immune cells into the thyroid gland.  Ref: 12864798 Clin Endocrinol (Oxf), 2003
- These data indicate that pathophysiological levels of Hcy can alter human monocyte function by upregulating MCP 1  [ **CCR2** ]   [ **CCL2** ]  and IL 8 expression and secretion via enhanced formation of intracellular ROS originated from NAD P H oxidase source via calmodulin or protein kinase C signaling pathways.  Ref: 12881478 Circ ResCirc Res, 2003
- The protein level of MCP 1  [ **CCR2** ]   [ **CCL2** ]  in the mild hypothermic subgroups was 8.7 7.6 ng.g tissue 1 6 h after reperfusion P <.  Ref: 12887739 Zhonghua Yi Xue Za Zhi, 2003
- Using cultured endothelial cells, we have shown that laminar shear stress causes a transient increase in monocyte chemotactic protein 1  [ **CCL2** ]  MCP 1  [ **CCR2** ]   [ **CCL2** ]  expression, which involves the Ras MAP kinase signaling pathway.  Ref: 12865076 Prog Biophys Mol Biol, 2003
- The expression of Ps of platelets and CD11b of leukocytes was determined by flowcytometry and the expression of MCP 1  [ **CCR2** ]   [ **CCL2** ]  mRNA of myocardial tissue was measured by RT PCR admission and at 8 60 min of reperfusion R5, R30, R60, R180, R360.  Ref: 12881868 Zhejiang Da Xue Xue Bao Yi Xue Ban, 2003
- These effects of Hcy were primarily mediated by reactive oxygen species ROS through NAD P H oxidase, because Hcy could upregulate the production of ROS and the inhibitors of protein kinase C, calmodulin, free radical scavengers, or NAD P H oxidase abolished Hcy induced ROS production and MCP 1  [ **CCR2** ]   [ **CCL2** ]  and IL 8 secretion in these cells.  Ref: 12881478 Circ ResCirc Res, 2003
- At all the concentrations tested, MOM was more effective than DEX in inhibiting ICAM 1 expression and MCP 1  [ **CCR2** ]   [ **CCL2** ]  release p< ; 0.05, each comparison.  Ref: 12877820 Pulm Pharmacol Ther, 2003
- Objective The role of monocyte chemotactic protein MCP 1  [ **CCR2** ]   [ **CCL2** ]  in human pulmonary and pleural tuberculosis TB was assessed by examining its production in clinical samples from patients with active pulmonary TB and tuberculous pleurisy TBP.  Ref: 12861395 Inflamm Res, 2003
- Angiotensin II induces several fibrogenic chemokines, namely monocyte chemoattractant protein 1  [ **CCL2** ]  MCP 1  [ **CCR2** ]   [ **CCL2** ]  and transforming growth factor beta.  Ref: 12882873 Diabetes Care, 2003
- The expression of MCP 1  [ **CCR2** ]   [ **CCL2** ]  positive cells almost coincided with the macrophages.  Ref: 12892039 J Cardiol, 2003
- The aim of the study was to evaluate the inhibitory effect of two corticosteroids, mometasone furoate MOM and dexamethasone DEX, respectively, on a variety of fibroblast functions DNA synthesis and proliferation, expression of adhesion molecules intercellular adhesion molecule 1 ICAM 1, CD54 and hyaluronic cellular adhesion molecule HCAM, CD44 and release of chemokines cytokines monocyte chemoattractant protein MCP 1  [ **CCR2** ]   [ **CCL2** ] , eotaxin, interleukin IL 6 and transforming growth factor TGF beta.  Ref: 12877820 Pulm Pharmacol Ther, 2003
- IL 8 and MCP 1  [ **CCR2** ]   [ **CCL2** ]  mRNA levels peaked within hours and were undetectable at 14 days.  Ref: 12866605 Vasc Med, 2003
- In contrast, staining for MCP 1  [ **CCR2** ]   [ **CCL2** ]  was little seen in the stellate vascular smooth muscle cells.  Ref: 12892039 J Cardiol, 2003
- The protein levels of IL 1beta and MCP 1  [ **CCR2** ]   [ **CCL2** ]  in the cortical ischemic core were measured by ELISA.  Ref: 12887739 Zhonghua Yi Xue Za Zhi, 2003
- In contrast, disturbed flow observed at branch points and simulated in step flow channels causes sustained activation of MCP 1  [ **CCR2** ]   [ **CCL2** ]  and the genes facilitating cell turnover and lipid accumulation.  Ref: 12865076 Prog Biophys Mol Biol, 2003
- Urinary MCP 1  [ **CCR2** ]   [ **CCL2** ]  levels decreased from 0.456 0.22 ng mg creatinine to 0.08 0.096 ng mg creatinine.  Ref: 12882873 Diabetes Care, 2003
- There were positive correlations between the concentration of IL 8 and the percentage of neutrophils p = 0.038 and between the concentration of MCP 1  [ **CCR2** ]   [ **CCL2** ]  and the percentage of lymphocytes p = 0.006.  Ref: 12872499 G Ital Med Lav ErgonG Ital Med Lav Ergon, 2003
- Oxygen supply modulates MCP 1  [ **CCR2** ]   [ **CCL2** ]  release in monocytes from young and aged rats decrease of MCP 1  [ **CCR2** ]   [ **CCL2** ]  transcription and translation is age related.  Ref: 12870648 Mol Cell Biochem, 2003
- We conclude that hyperoxia is an important regulator of MCP 1  [ **CCR2** ]   [ **CCL2** ]  release and support the hypothesis that increased % of O2 may serve to initiate MCP 1  [ **CCR2** ]   [ **CCL2** ]  production which then serves to recruit and regulate the distribution of mononuclear cells to the sites of inflammation.  Ref: 12870648 Mol Cell Biochem, 2003
- The inhibition of PNF by AsA was associated with decreased intra islet levels of inflammation related molecules IL 1, TNF alpha, iNOS, COX 2 and chemokines MCP 1  [ **CCR2** ]   [ **CCL2** ]  and MIP 3alpha.  Ref: 12879250 DiabetologiaDiabetologia, 2003
- In this study we evaluated if the ability of monocytes to produce MCP 1  [ **CCR2** ]   [ **CCL2** ]  under basal conditions or after stimulation with lipopolysaccharide LPS or phytohaemagglutinin PHA was differently affected by exposure to hyper or hypoxic conditions in young and aged rats.  Ref: 12870648 Mol Cell Biochem, 2003
- MgSO 4 inhibited the expression of Ps of platelets and MCP 1  [ **CCR2** ]   [ **CCL2** ]  mRNA of myocardial tissue and decreased serum Ca 2 concentration, but did not affect the expression of CD11b of leukocytes.  Ref: 12881868 Zhejiang Da Xue Xue Bao Yi Xue Ban, 2003
- In parallel, the response to  **CCL2**  MCP 1  [ **CCR2** ]   [ **CCL2** ]  was lost after infection with active virus Neither a  **CCL2**  MCP 1  [ **CCR2** ]   [ **CCL2** ]  induced shift of intracellular calcium concentrations nor the chemotactic response to  **CCL2**  MCP 1  [ **CCR2** ]   [ **CCL2** ]  was detectable.  Ref: 12885942 J Leukoc Biol, 2003
- We demonstrated the spatial pattern of MCP 1  [ **CCR2** ]   [ **CCL2** ]  mRNA levels through in situ mRNA hybridization.  Ref: 12866605 Vasc Med, 2003
- OBJECTIVE To investigate the effect of ischemic reperfusion I R on the expression of P selectin Ps of platelets, CD11b of leukocytes, monocyte chemotactic protein 1  [ **CCL2** ]  MCP 1  [ **CCR2** ]   [ **CCL2** ]  mRNA of myocardial tissue and the protective role of MgSO 4.  Ref: 12881868 Zhejiang Da Xue Xue Bao Yi Xue Ban, 2003
- The effects of resistin on expression of vascular cell adhesion molecule VCAM 1 and monocyte chemoattractant chemokine MCP 1  [ **CCR2** ]   [ **CCL2** ]  were studied in addition to CD40 receptor, CD40 ligand induced MCP 1  [ **CCR2** ]   [ **CCL2** ]  expression, and tumor necrosis factor receptor associated factor 3 TRAF3, an inhibitor of CD40 signaling.  Ref: 12874180 CirculationCirculation, 2003
- ACE Inhibitors Improve Diabetic Nephropathy Through Suppression of Renal MCP 1  [ **CCR2** ]   [ **CCL2** ] .  Ref: 12882873 Diabetes Care, 2003
- RESULTS Expression of MCP 1  [ **CCR2** ]   [ **CCL2** ] , RANTES, MIP 1alpha, MIP 1beta, IP 10 and Mig was demonstrated in all Hashimoto s and most Graves thyroid specimens but very little expression was detected in the nonautoimmune goitre samples.  Ref: 12864798 Clin Endocrinol (Oxf), 2003
- CONCLUSIONS Both MCP 1  [ **CCR2** ]   [ **CCL2** ]  and the M CSF c fms system are involved in the atherogenesis of de novo lesions.  Ref: 12892039 J Cardiol, 2003
- Neutralizing antibodies against human RANTES, MIP 1alpha, MIP 1beta, alpha interferon IFN alpha, IFN beta, IFN gamma, interleukin 4 IL 4, IL 10, IL 13, IL 16, MCP 1  [ **CCR2** ]   [ **CCL2** ] , MCP  [ **CCL2** ]  3, tumor necrosis factor alpha TNF alpha, or TNF beta failed to reverse the HIV 1 suppressive activity.  Ref: 12885891 J VirolJ Virol, 2003
- Up regulation of IL 6, IL 8, and MCP 1  [ **CCR2** ]   [ **CCL2** ]  mRNA expression and production by the virus in combination with opaque S. pneumoniae was two to threefold higher than that induced by the virus combined with the transparent S. pneumoniae variant.  Ref: 12874304 Infect Immun, 2003
- However, both S. pneumoniae opacity variants increased mRNA expression of MIP 1alpha, MIP 1beta, IL 6, and MCP 1  [ **CCR2** ]   [ **CCL2** ]  in HMEE cells activated by a prior influenza A virus infection compared to levels in cells treated with either agent alone.  Ref: 12874304 Infect Immun, 2003
- High levels of RANTES CCL5, MCP 1  [ **CCR2** ]   [ **CCL2** ]   **CCL2** , IP 10 CXCL10, and Mig CXCL9 simultaneously with mononuclear cell infiltration in the lungs was found.  Ref: 12875978 Am J Pathol, 2003
- We studied polymorphisms in the genes for monocyte chemoattractant protein 1  [ **CCL2** ]  MCP 1  [ **CCR2** ]   [ **CCL2** ]  and CC chemokine receptor CCR 2  [ **CCR2** ]  in 171 Parkinson s disease PD patients and 340 controls.  Ref: 12889089 Mov Disord, 2003
- We analyzed the mRNA levels of the chemokines monocyte chemoattractant protein 1  [ **CCL2** ]  MCP 1  [ **CCR2** ]   [ **CCL2** ]  and interleukin 8 IL 8 through semi quantitative polymerase chain reaction.  Ref: 12866605 Vasc Med, 2003
- IL 8 and MCP 1  [ **CCR2** ]   [ **CCL2** ]  mRNA levels peaked within hours but were still detectable 14 days post injury.  Ref: 12866605 Vasc Med, 2003
- This effect was also detected in a second PD group, suggesting a possible involvement of MCP 1  [ **CCR2** ]   [ **CCL2** ]  in PD.  Ref: 12889089 Mov Disord, 2003
- However, spontaneous, LPS or PHA induced MCP 1  [ **CCR2** ]   [ **CCL2** ]  production was up regulated by exposure to hyperoxic conditions in both young 62 9 02 8 pg ml, respectively and aged rats 79 12 17 10 pg ml, respectively.  Ref: 12870648 Mol Cell Biochem, 2003
